# Supplementary material for: Fear of becoming pregnant among female healthcare students in Spain
Source: PeerJ. 2015 Aug 25;3:e1200. doi: 10.7717/peerj.1200 (PMC4556155; doi:10.7717/peerj.1200)
Supplement: Table S1 [file peerj-03-1200-s002.doc]

| **General items** | **Cuestiones generales** |
| --- | --- |
| A Age:  B Marital Status:  Single  Married  Widowed  Separated  Divorced  Other  C Sexual Orientation:  Bisexual  Heterosexual  Homosexual  Other  D Method of contraception you use: 1 condoms, 2 oral contraceptives, 3 none, 4 *reverse/withdrawal*, 5 IUD, 6 vaginal ring, 7 patch, 8 several, 9 abstinence.  E Are you in a stable relationship? Yes No  F Do you suffer from any illness?  Yes No  G Which?  Since when?  H Do you take any treatment for this? Yes No  I Which?  J Does your partner suffer from any illness? Yes No  K Which?  Since when?  L Does your partner take any treatment for this? Yes No  Which? | A Edad:  B Estado Civil:  Soltera  Casada  Viuda  Separada  Divorciada  Otros  C Orientación sexual:  Bisexual  Heterosexual  Homosexual  Otro  D Método anticonceptivo que utiliza: 1 preservativo, 2 anticonceptivo oral, 3 ninguno, 4 *marcha atrás*, 5 DIU, 6 anillo vaginal, 7 parches, 8 varios, 9 abstinencia.  E ¿Tiene pareja estable? Sí No  F ¿Padece alguna enfermedad?  Sí No  G ¿Cual?  ¿Desde cuándo?  H ¿Toma algún tratamiento para esta? Sí No  I ¿Cuál?  J ¿Padece su pareja alguna enfermedad? Sí No  K ¿Cuál?  ¿Desde cuándo?  L ¿Toma algún tratamiento para esta? Sí No  ¿Cuál? |
| **The following questions refer to the last three months.** | **Las preguntas siguientes están referidas a los últimos 3 meses.** |
| **General sexual items** | **Ítems generales de sexualidad** |
| 1. Suppose the following is a scale of SEXUAL APPETITE. Where would you place yourself on it?  A little 1 2 3 4 5 6 7 A lot | 1. Suponga que la siguiente es una escala de APETENCIA SEXUAL. ¿Dónde se situaría usted en ella?  Poca 1 2 3 4 5 6 7 Mucha. |
| **Masturbation items** | **Ítems de masturbación** |
| 2. Have you masturbated on any occasion? Yes No  3. How frequently?  5-7 times/week  3-4 times/week  1-2 times/week  2-3 times/month  once/month  Less than once/month  4. Suppose the following is a scale of sexual EXCITATION. Where would you place yourself on it, when practicing this type of sexual activity?  A little 1 2 3 4 5 6 7 A lot  5. How much satisfaction does this type of sexual activity give you?  Unsatisfied 1 2 3 4 5 6 7 Satisfied  6. How frequently do you have orgasms while practicing this type of sexual activity?  Never 1 2 3 4 5 6 7 Always | 2. ¿Se ha masturbado en alguna ocasión? Sí No  3. ¿Con qué frecuencia?  5-7 veces/semana  3-4 veces/semana  1-2 veces/semana  2-3 veces/mes  1 vez/mes  Menos 1 vez mes  4.- Suponga que la siguiente es una escala de EXCITACION sexual. ¿Dónde se situaría usted al practicar este tipo de actividad sexual?  Poca 1 2 3 4 5 6 7 Mucha  5. ¿Qué satisfacción le produce este tipo de actividad sexual?  Insatisfecho 1 2 3 4 5 6 7 Satisfecho  6. ¿Con qué frecuencia tiene orgasmos al practicar este tipo de actividad sexual?  Nunca 1 2 3 4 5 6 7 Siempre |
| **Non vaginal intercourse items** | **Ítems de relaciones sexuales no vaginales** |
| 7. How frequently do you have sexual relations with your partner, excluding vaginal intercourse? (This includes manual stimulation, oral stimulation, etc.)  5-7 times/week  3-4 times/week  1-2 times/week  2-3 times/month  once/month  Never  8. Suppose that the following is a scale of sexual EXCITATION. Where would you place yourself when practicing this type of sexual activity?  A little 1 2 3 4 5 6 7 A lot  9. What satisfaction does this type of sexual activity produce for you?  Unsatisfied 1 2 3 4 5 6 7 Satisfied  10. How frequently do you have orgasms while practicing this type of sexual relation?  Never 1 2 3 4 5 6 7 Always | 7. ¿Con qué frecuencia tiene relaciones sexuales con su pareja, excluyendo el coito? (Se incluye estimulación manual, oral, etc.)  5-7 veces/semana  3-4 veces/semana  1-2 veces/semana  2-3 veces/mes  1 vez/mes  Nunca  8. Suponga que la siguiente es una escala de EXCITACION sexual. ¿Dónde se situaría usted al practicar este tipo de actividad sexual?  Poca 1 2 3 4 5 6 7 Mucha  9. ¿Qué satisfacción le produce este tipo de actividad sexual?  Insatisfecho 1 2 3 4 5 6 7 Satisfecho  10. ¿Con qué frecuencia tiene orgasmos al practicar este tipo de relaciones sexuales?  Nunca 1 2 3 4 5 6 7 Siempre |
| **Vaginal intercourse items** | **Ítems de relaciones sexuales vaginales** |
| 11. How frequently do you have sexual intercourse with your partner?  5-7 times/week  3-4 times/week  1-2 times/week  2-3 times/month  once/month  Never  12. Suppose that the following is a scale of sexual EXCITATION. Where would you place yourself when practicing this type of sexual activity?  A little 1 2 3 4 5 6 7 A lot  13. What satisfaction does this type of sexual activity produce for you?  Unsatisfied 1 2 3 4 5 6 7 Satisfied  14. How frequently do you have orgasms while practicing this type of sexual relations?  Never 1 2 3 4 5 6 7 Always | 11. ¿Con qué frecuencia tiene relaciones sexuales de coito con su pareja?  5-7 veces/semana  3-4 veces/semana  1-2 veces/semana  2-3 veces/mes  1 vez/mes  Nunca  12. Suponga que la siguiente es una escala de EXCITACION sexual. ¿Dónde se situaría usted al practicar este tipo de actividad sexual?  Poca 1 2 3 4 5 6 7 Mucha  13. ¿Qué satisfacción le produce este tipo de actividad sexual?  Insatisfecho 1 2 3 4 5 6 7 Satisfecho  14. ¿Con qué frecuencia tiene orgasmos al practicar este tipo de relaciones sexuales?  Nunca 1 2 3 4 5 6 7 Siempre |
| **Orgasm items** | **Ítems de orgasmos** |
| 15. How do you normally have orgasms?  During vaginal intercourse  Through fantasies and daydreams  By stimulation from my partner  By self stimulation  Through various of the previous methods  I don’t have orgasms By other methods | 15. ¿Cómo tienen lugar normalmente sus orgasmos?  Durante el coito vaginal  Por fantasías y ensoñaciones  Por estimulación de mi pareja  Por auto-estimulación  Por varios de los métodos anteriores  No tengo orgasmos  Por otros métodos |
| **Changes in sex life items** | **Ítems de cambios en la vida sexual** |
| 16. What aspects of your sex life would you change?  Increase the frequency of sexual relations  To have the same sexual appetite as my partner  To increase my capacity to have orgasms  To eliminate the fear of pregnancy  To increase my partner’s capacity to delay orgasm  More variety (hour of the day, position, etc.)  Nothing  Other__ Which? | 16. ¿Qué aspectos de su vida sexual cambiaría usted?  Aumentaría la frecuencia de las relaciones sexuales  Tener la misma apetencia sexual que mi pareja  Aumentar la capacidad para tener orgasmos  Eliminar el miedo al embarazo  Aumentar la capacidad de mi pareja para demorar el orgasmo  Mayor variedad (hora del día, posición, etc)  Ninguno  Otros__ ¿Cuáles? |
| **Sexual partner items** | **Ítem de la pareja sexual** |
| 17. In relation to your partner, indicate the things that you find unpleasant in your sexual relationship.  Shows little enthusiasm  Penis is too small  Has difficulty maintaining an erection  Too slow in ejaculating  Wishes to remove his penis too quickly Wants intercourse too frequently  Penis is too big  Has difficulty achieving erection  Cannot always ejaculate  Ejaculates too quickly  Wishes to sleep after intercourse  Rarely desires intercourse  Too sexually demanding  Is not caring enough during intercourse  Wants to do things that do not seem natural for me  Cares little for my sexual satisfaction  Does not stimulate or caress me enough before intercourse  18. Do you refuse to have intimate sexual relations when your partner wants it?  Very frequently  Frequently  Sometimes  Rarely  Never  19. If you refuse to have intimate sexual relations with your partner, how does he react?  Insistent or irritable  Annoyed, although not for long time  Considerate and pleasant  20. How do you feel when you are naked in front of your partner?  Uncomfortable 1 2 3 4 5 6 7 Comfortable  21. What do you believe is your partner’s level of satisfaction in your sexual relationship?  Unsatisfied 1 2 3 4 5 6 7 Very satisfied  22. In regards to you, what things do you believe your partner finds unpleasant in your sexual relationship?  I show little enthusiasm  Vagina is too small  I never have an orgasm  I orgasm too quickly  I desire intercourse too frequently  I am rarely caring during sex  I like to practice unnatural things  Vagina is too big  I am too slow at attaining an orgasm  I want to sleep after I have had an orgasm  I rarely want sex  I do not stimulate or caress him enough before sex. | 17. En relación a su pareja, señale las cosas que encuentra desagradables en sus relaciones sexuales:  Muestra poco entusiasmo  Pene demasiado pequeño  Tiene dificultades en mantener la erección  Demasiado lento en eyacular  Desea retirar su pene con demasiada rapidez  Desea el coito con demasiada frecuencia  Pene demasiado grande  Tiene dificultades para lograr la erección  No siempre puede eyacular  Eyacula rápidamente  Desea dormir tras el coito  Desea el coito raramente  Es demasiado exigente sexualmente  Es muy poco cariñoso durante el coito  Desea realizar cosas que no me parecen naturales  Se preocupa poco por mi satisfacción sexual  No me estimula ni acaricia lo suficiente antes del coito  18. ¿Rehúsa tener relaciones sexuales íntimas cuando su pareja lo desea?  Muy frecuentemente  Frecuentemente  Algunas veces  Raramente  Nunca  19. Si rehúsa tener relaciones sexuales íntimas con su pareja, ¿cómo reacciona?  insistente o irritable  Disgustada, aunque no por mucho tiempo  Considerada y agradable  20. ¿Cómo se siente al estar desnuda ante su pareja?  Incómoda 1 2 3 4 5 6 7 Cómoda  21. ¿Cuál cree que es el grado de satisfacción de su pareja en sus relaciones sexuales?  Insatisfecho 1 2 3 4 5 6 7 Muy satisfecho  22. En relación a usted, ¿qué cosas cree que encuentra desagradables su pareja en sus relaciones sexuales?  Muestro poco entusiasmo  Vagina demasiado pequeña  Nunca tengo un orgasmo  Tengo un orgasmo muy rápidamente  Deseo el coito con demasiada frecuencia  Soy muy poco cariñosa en el coito  Me gusta practicar cosas poco naturales  Vagina demasiado grande  Soy demasiado lenta para tener un orgasmo  Deseo dormir después de tener un orgasmo  Deseo el coito raramente  No le estimulo y acaricio suficientemente antes del coito |
